# Optimal cut-off age for AJCC staging system in Middle Eastern differentiated thyroid cancer

**DOI:** 10.3389/fendo.2025.1494154

**Published:** 2025-11-24

**Authors:** Sandeep Kumar Parvathareddy, Abdul K. Siraj, Zeeshan Qadri, Padmanaban Annaiyappanaidu, Nabil Siraj, Saif S. Al-Sobhi, Fouad Al-Dayel, Khawla S. Al-Kuraya

**Affiliations:** 1 Human Cancer Genomic Research, Research Centre, King Faisal Specialist Hospital and Research Centre, Riyadh, Saudi Arabia; 2 Department of Surgery, King Faisal Specialist Hospital and Research Centre, Riyadh, Saudi Arabia; 3 Department of Pathology, King Faisal Specialist Hospital and Research Centre, Riyadh, Saudi Arabia

**Keywords:** differentiated thyroid cancer, AJCC, optimal age, disease-specific survival, Harrel’s C-index

## Abstract

**Background:**

Age cut-off of 55 years has been included in the eighth edition of the American Joint Committee on Cancer (AJCC) TNM staging, since it led to better prediction of disease-specific survival (DSS) of patients with differentiated thyroid cancer (DTC). However, optimal age cut-off in DTC patients from Middle Eastern ethnicity has not been fully explored.

**Methods:**

We retrospectively analyzed a large cohort of 1721 adult DTC patients. The optimal age cut-off value was determined using several age cut-offs (between 20 and 85 years) to assess DSS. Harrel’s C-Index, Akaike information criterion (AIC) and Bayesian Information Criterion (BIC) were used to assess statistical model performance of the TNM staging system (eighth edition), with different age cut-offs for prediction of DSS.

**Results:**

The median age of patients at diagnosis was 39.9 years (inter-quartile range 31.0 – 51.7 years) and 75.5% (1299/1721) were female. Median follow up was 9.3 years and 10 years DSS was 97.1%. For DTC overall, an age cut-off of 50 years had the best statistical model performance. On receiver operating characteristic curve analysis, the optimal age cut-off for prediction of DSS was 50.5 years (area under the curve = 0.872, p < 0.0001).

**Conclusion:**

In this large cohort of Middle Eastern DTC patients, an age cut-off of 50 years was more appropriate for TNM staging to achieve better predictability for DSS. Therefore, implementation of different age cut-off for DTC in Middle Eastern patients could improve the predictive value for TNM staging system, allowing for better therapeutic and surveillance approach for these patients.

## Introduction

Differentiated Thyroid Cancer (DTC), a common endocrine malignancy, has witnessed a significant rise in global incidence over the past few decades (1, 2). Despite its indolent nature and favorable prognosis, the clinical course of DTC varies extensively, ranging from an asymptomatic disease course to aggressive forms associated with high morbidity and mortality (3–5). The staging and subsequent management of DTC presents a unique set of challenges due to the heterogeneous clinical behavior of the disease (6). Accurate risk stratification is critical for optimizing patient management, guiding therapeutic decisions, and estimating the disease-specific survival (DSS).

The American Joint Committee on Cancer (AJCC) TNM staging system has been universally adopted to predict the prognosis of DTC patients (7). The eighth edition of the AJCC TNM staging system has incorporated an age cut-off of 55 years, based on the observation that this cut-off allowed for a more precise prediction of DSS (8–10). Although the AJCC staging system is globally coordinated through national committees, the empirical foundation for the 8th edition’s 55-year age cut-off originated mainly from large North American and European cohorts (9, 11). Thus, validation in ethnically distinct populations such as the Middle East remains warranted. This age cut-off, however, is predominantly derived from data on Western populations, raising questions about its generalizability to other ethnic groups. Subsequent validation studies in diverse populations, including South Korea, Italy, Poland, Argentina, Japan and Netherlands, have generally supported the clinical utility of this cut-off, though regional variations in age-related risk persist (10, 12–16).

Notably, the Middle Eastern population, which has a high prevalence of DTC (17) and more aggressive disease characteristics (18–21), may demonstrate a different age-related risk profile in DTC. Several regional series have reported younger age at diagnosis and higher frequencies of multifocality, extrathyroidal extension, lymph-node metastasis and ATA high risk in Middle Eastern DTC patients (18, 19, 21–24) compared with Western cohorts (11, 25–28). These patterns persist despite comparable healthcare access, supporting the likelihood of population-specific biological or genetic determinants of tumor behavior. However, the optimal age cut-off for TNM staging in Middle Eastern DTC patients remains an unexplored area of research.

Recognizing the potential implications of tailoring the age cut-off for AJCC TNM staging to the specific characteristics of Middle Eastern DTC patients, we embarked on this study. The primary objective of our investigation was to identify the optimal age cut-off for predicting DSS in this patient population. We believe that establishing a region-specific age cut-off could enhance the predictive value of the AJCC TNM staging system, leading to improved therapeutic planning and surveillance strategies for Middle Eastern patients with DTC.

## Materials and methods

### Patient selection

One thousand seven-hundred and twenty-one consecutive unselected adult DTC patients (≥ 18 years) diagnosed between 1988 and 2018 at King Faisal Specialist Hospital and Research Centre (KFSHRC, Riyadh, Saudi Arabia) were included in the study. Cases were identified based on clinical history followed by fine needle aspiration cytology for confirmation. Final inclusion in the study cohort required histopathological confirmation following surgical resection. All histopathologic slides were reviewed by a dedicated endocrine pathology team at KFSHRC, with consensus confirmation for any discordant cases by two senior pathologists to ensure uniform diagnostic standards. The Institutional Review Board of the hospital approved this study and the Research Advisory Council (RAC) provided waiver of consent under project RAC # 2211–168 and # 2110 031.

### Clinico-pathological data

Baseline clinico-pathological data were collected from case records and have been summarized in **Table 1**. Patients were classified using the eighth edition of the TNM staging system (7). Furthermore, reclassification was performed applying different age cut-offs, using the histopathological criteria from the eighth edition of the TNM system. For this purpose, we investigated age cutoffs at 5-year increments from 20 years up to 85 years. Additionally, we also investigated 1-year increments between 35 and 55 for sensitivity analysis. For the two-step age cut-off analysis, lower age threshold was analyzed from 20 up to 60 years with 5-year increments and upper age threshold was analyzed from 40 up to 60 years with 5-year increments. Patients having an age below the lower threshold were classified as stage I, age between lower and upper threshold as stage II and at or above the upper threshold as stage IV. DSS was used as the study endpoint and defined as the time from diagnosis to death due to DTC progression.

**Table 1:** Clinico-pathological characteristics of the study cohort.

Clinico-pathological characteristic	Total (n = 1721)
Age (years)	
Median (IQR), years	39.9 (31.0 – 51.7)
Gender	
Female	1299 (75.5)
Male	422 (24.5)
Histologic subtype	
Papillary thyroid carcinoma	1621 (94.2)
Follicular thyroid carcinoma	100 (5.8)
Tumor laterality	
Unilateral	1184 (68.8)
Bilateral	529 (30.7)
Unknown	8 (0.5)
Multifocality	
Yes	821 (47.7)
No	892 (51.8)
Unknown	8 (0.5)
Extrathyroidal extension	
Present	674 (39.2)
Absent	1047 (60.8)
Lymphovascular invasion	
Present	477 (27.7)
Absent	1244 (72.3)
pT	
T1	670 (38.9)
T2	556 (32.3)
T3	374 (21.7)
T4	120 (7.0)
Unknown	1 (0.1)
pN	
N0	723 (42.0)
N1	777 (45.2)
Nx	221 (12.8)
pM	
M0	1636 (95.1)
M1	85 (4.9)
TNM Stage	
I	1435 (83.4)
II	193 (11.2)
III	24 (1.4)
IV	62 (3.6)
Unknown	7 (0.4)

### Statistical analysis

The relative prognostic performance of the TNM staging system with different age cut-offs was evaluated in DTC using the concordance index (Harrell’s C-index) (29, 30), Akaike information criterion (AIC) (31) and Bayesian information criterion (BIC) (32). In addition, a sensitivity analysis limited to papillary thyroid cancer (PTC) cases only (n=1621) was also conducted using C-index, AIC and BIC. The C-index commonly is used to evaluate risk models in survival analysis. It measures the discriminative power of a model and is a measure of goodness of fit. A model with perfect predictive capacity (sensitivity and specificity of 100%) would have a Harrell’s C-index of 1.00; a category that exhibited a higher Harrell’s C-index was considered to exhibit a more accurate predictive capacity. Furthermore, the AIC and BIC measure the relative quality of a statistical model. The model with the lowest AIC and BIC values is considered to be the best model for predicting outcomes. C-index, AIC and BIC were calculated using R version 4.0.1.

Receiver operating characteristic (ROC) curve analysis was also used to determine the age cut-off, with DSS as the outcome. We calculated the area under curve (AUC), sensitivity and specificity. ROC curve analysis was performed using MedCalc software, version 10.4.7.0 for Windows (MedCalc, Ostend, Belgium).

## Results

### Patient and tumor characteristics

Median age of the study population was 39.9 years (inter-quartile range: 31.0 – 51.7 years), with a male to female ratio of 1:3. The majority of tumors were PTC (94.2%; 1621/1721). 30.7% (529/1721) of tumors were bilateral and 47.7% (821/1721) were multifocal. Extrathyroidal extension was noted in 39.2% (674/1721). Based on the eighth edition of TNM staging, 83.4% (1435/1721) of DTCs were stage I, 11.2% (193/1721) stage II, 1.4% (24/1721) stage III and 3.6% (62/1721) stage IV (**Table 1**). Median follow up was 9.3 years and 36 patients died due to disease-specific causes. Ten years DSS rate was 97.1%.

### Determination of age cut-off with best prognostic performance

**Figure 1:**
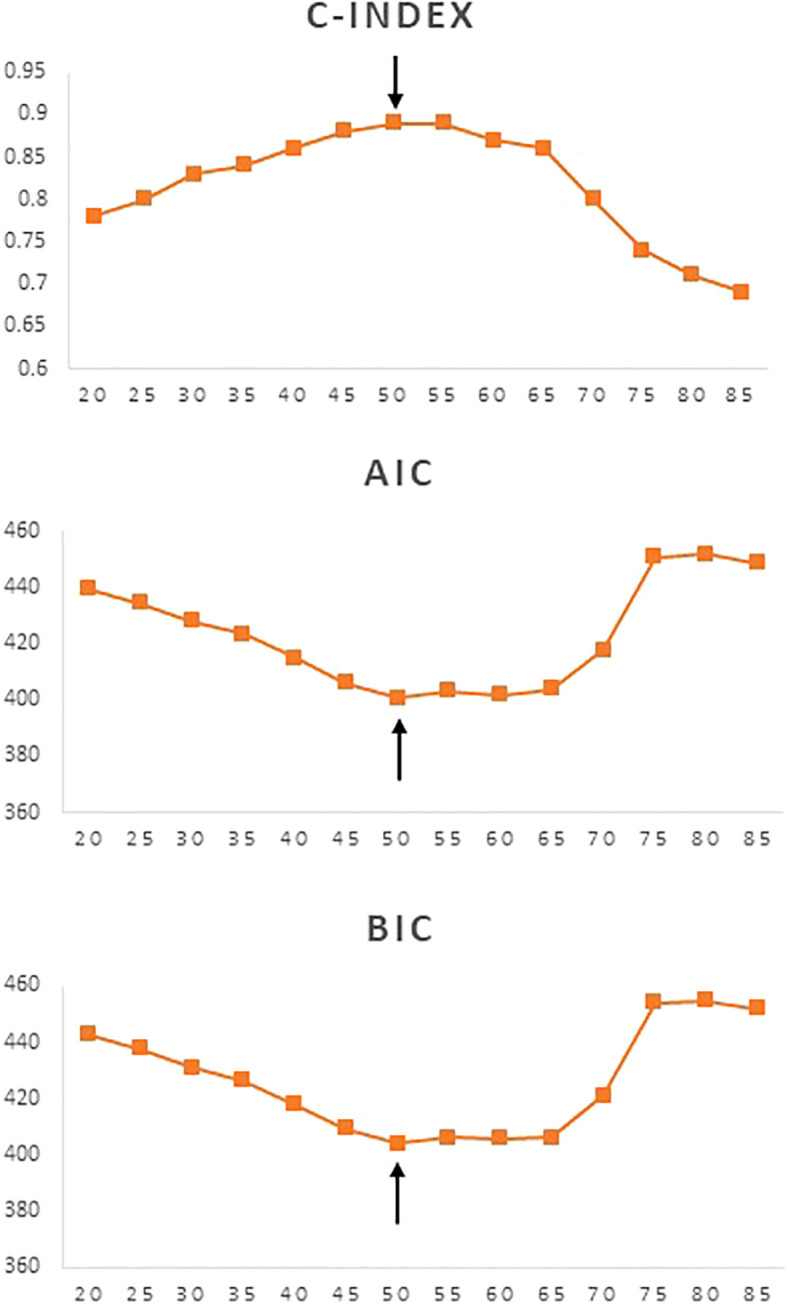
Statistical model performance for different age cut-offs (5-year increments) using C-index, Akaike information criterion (AIC), and Bayesian information criterion (BIC), with the arrows showing optimal age cut-off.

**Figure 2:**
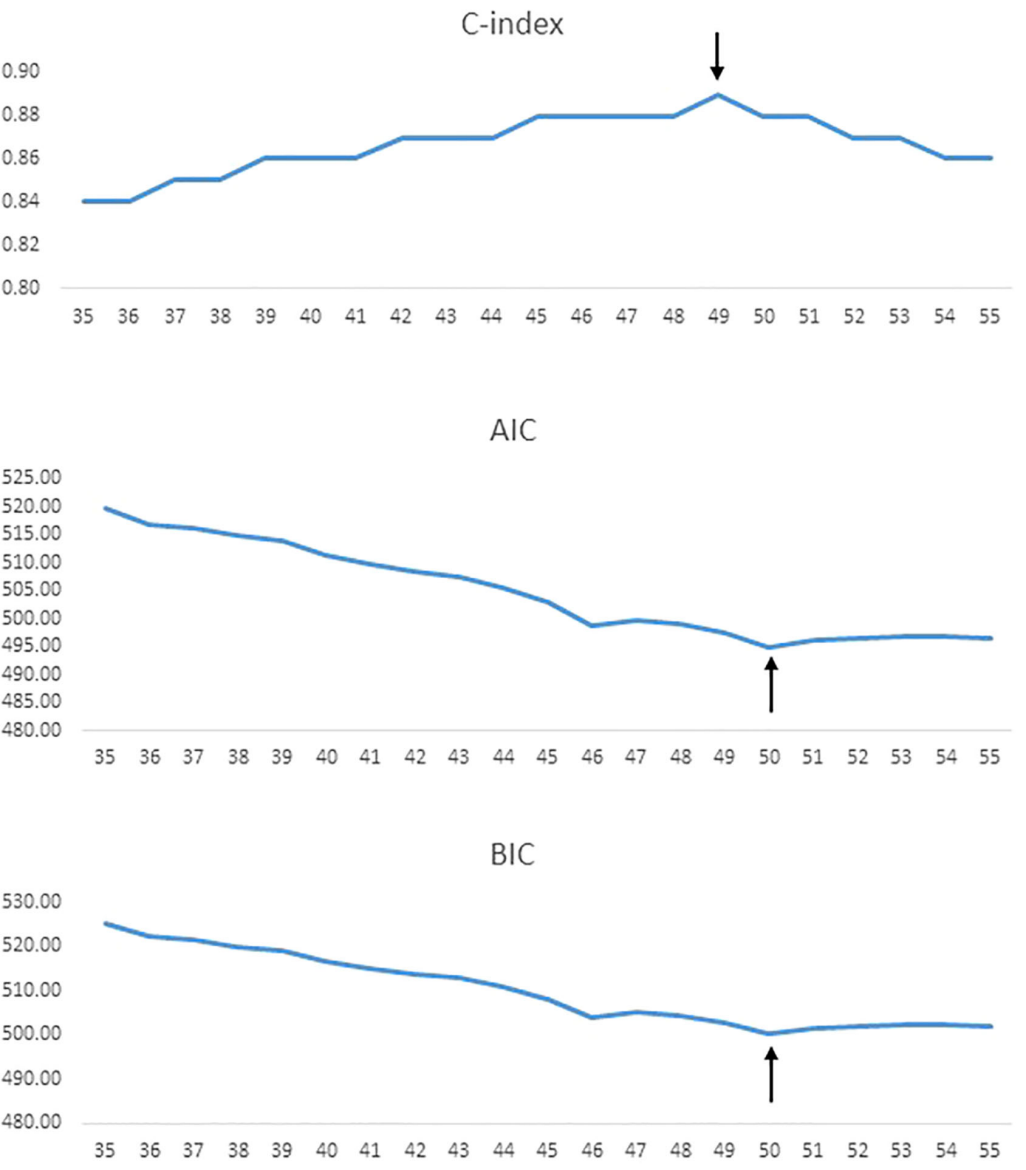
Statistical model performance for different age cut-offs (1-year increments) using C-index, AIC, and BIC, with the arrows showing optimal age cut-off.

Using 5-year increments for age cutoffs, the highest C-index was identified for an age cut-off of 50 years for DTC (C-index = 0.89). Similarly, lowest AIC and BIC values were seen at an age cut-off of 50 years for DTC (**Figure 1**). To further determine the exact age cut-off with the best statistical performance, we used 1-year increments. For DTC, the highest C-index was established with an age cutoff of 49 years (C-index = 0.89), whereas lowest AIC and BIC values (494.9 and 500.3, respectively) were seen at 50 years (**Table 2**, **Figure 2**). For comparison, the current 55-year cut-off yielded a C-index of 0.88, AIC of 496.6, and BIC of 502.0, which were inferior to the performance metrics at the 50-year cut-off. A sensitivity analysis restricted to PTC cases (n = 1621) showed consistent results, with 50 years remaining the optimal cut-off (**Table 2**).

**Table 2:** Model performance using one-step and two-step age cut-offs for disease-specific survival.

Age cut-off	Differentiated thyroid cancer (DTC)			Papillary thyroid cancer (PTC)		
	C-index	AIC	BIC	C-index	AIC	BIC
50 years	0.89	494.9	500.3	0.89	400.8	404.0
55 years	0.88	496.6	502.0	0.88	403.1	406.3
40 and 50 years (i.e., <40, 40–50 and ≥50)	0.86	496.5	503.5	0.87	403.0	405.7

**Figure 3:**
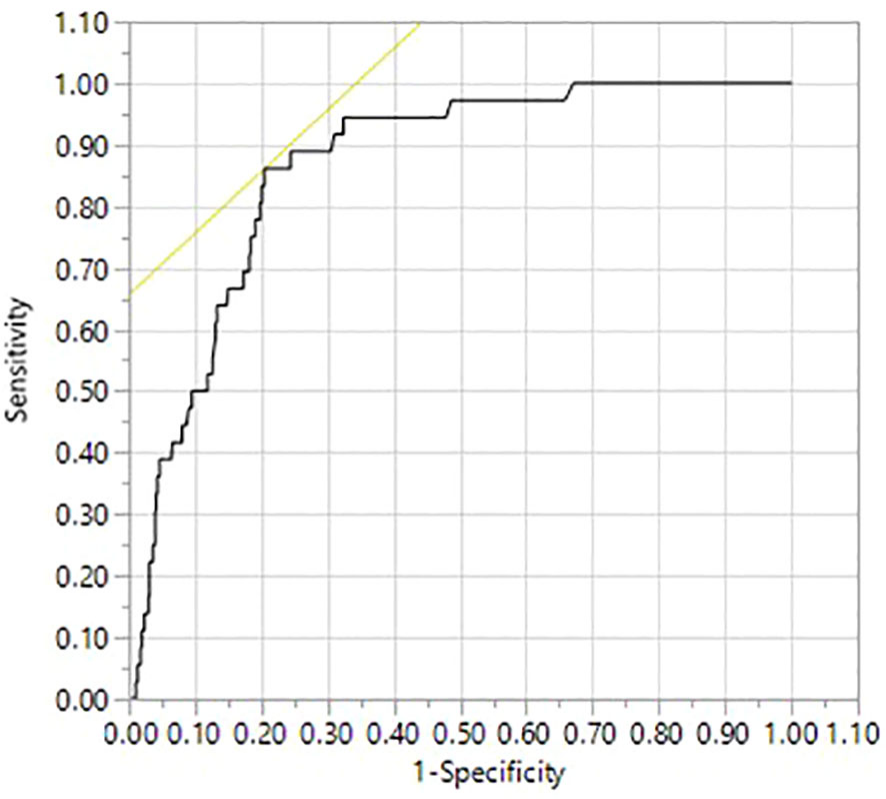
Receiver operating characteristic (ROC) curve for optimal age cut-off. An age cut-off of 50.5 years predicted disease-specific survival (DSS) with a sensitivity of 86.1%, specificity of 75.7%, and area under cover (AUC) of 0.872 (p < 0.0001).

To further corroborate our findings, we employed the ROC curve to determine the ideal age cut-off to predict DSS. We found that an age cut-off of 50.5 years was related to DSS with AUC of 0.872, sensitivity of 86.1% and specificity of 75.7% (p < 0.0001; **Figure 3**).

Additionally, we tested several two-step age stratification models. Among them, the model with <40, 40–50, and >50 years demonstrated the most favorable performance. However, this model did not outperform the single 50-year cut-off in terms of Harrell’s C-index, AIC, and BIC values (**Table 2**).

### Survival analysis based on stage using the new age cut-off

**Figure 4:**
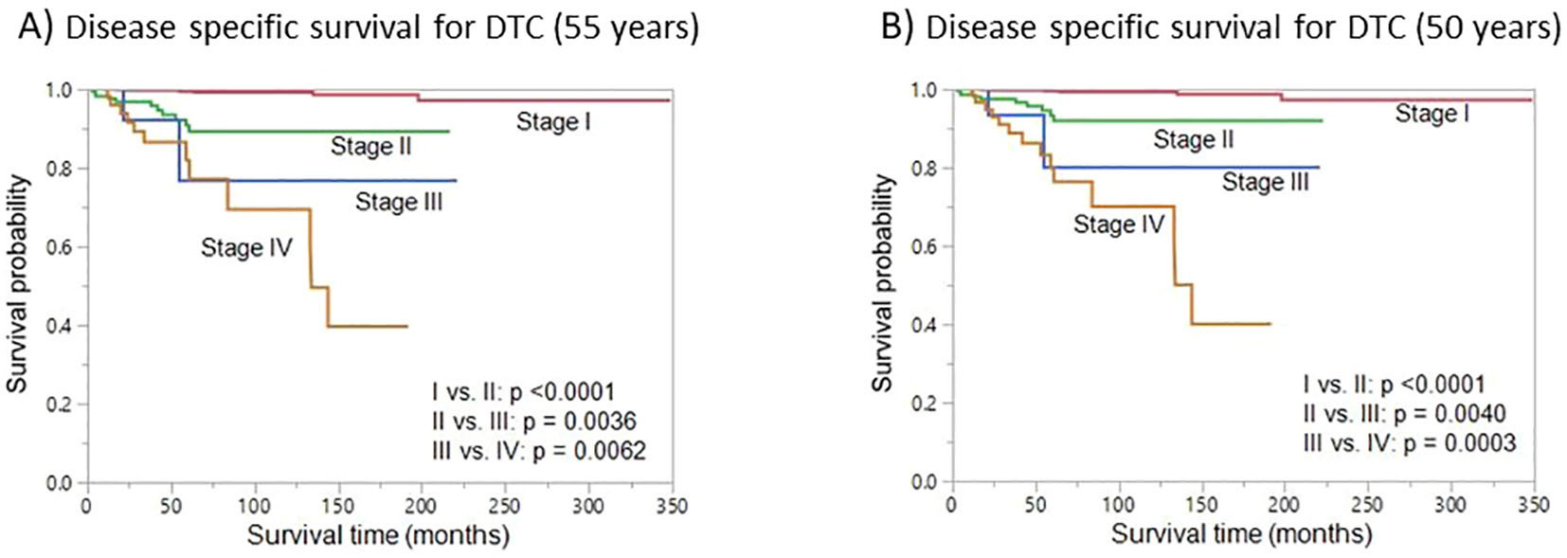
Disease-specific survival (DSS). Kaplan-Meier survival curve showing the DSS for different stages using the original 55-year age cutoff **(A)** and the cutoff with the best statistical performance (50 years) **(B)**.

We next sought to analyze the prognostic implications of using the new age cut-off (50 years) for re-staging. Lowering the age cutoff to 50 years for TNM staging resulted in a lower number of patients in stage I; these patients were redistributed over the other three higher stages (II – IV). From the original age cutoff toward the best identified cutoffs, 4.4% (75/1721) of DTC patients migrated to a higher stage. On Kaplan-Meier survival curve analysis, we found that DSS was similar for 55 and 50 years age cut-off, with regards to different tumor stages (**Figure 4**).

## Discussion

Our comprehensive analysis of a large cohort, over a span of almost thirty years, offers an opportunity to study the influence of age on DTC prognosis in the Middle Eastern ethnicity. The disease predominantly impacts females, as evidenced by the 75% representation within our study group. Intriguingly, the median age of diagnosis was considerably younger than global benchmark of 55 years, registering at 39.9 years (33–35). This deviation itself challenges the universal relevance of the globally accepted age cut-off of the eighth edition of the AJCC TNM system.

In this study, we showed that when employing histopathological staging from the eighth edition of the AJCC TNM classification of disease stage with respect to DSS, 50 years was a better predictor, rather than 55 years currently being used in staging system. To ensure robustness in our findings, we employed statistical metrics such as Harrel’s C Index, AIC, and BIC. Our analysis revealed that 50 years age threshold exhibited superior statistical model accuracy. This finding was further corroborated by receiver operating characteristic curve analysis, which identified 50.5 years as the ideal age cut-off for predicting DSS, showcasing a significant area under the curve (AUC = 0.872).

There are many studies from several ethnic population showing that the age of 55 years outperformed the previous cut-off of 45 years (9, 11, 12, 15, 36). However, all these studies have applied the seventh instead of the eighth TNM edition used in our study. Our study aligns with a recent large European population of DTC (37). When employing the histopathological criteria of the TNM system (eighth edition), they identified similar age cut-off of 50 years to predict DSS. This study concludes that implementing a lower age cut-off, such as 50 years, in the TNM staging system improves predictability of DSS in Middle Eastern patients with DTC. This underscores the importance of considering population-specific factors when determining age cut-off for risk stratification and treatment planning in DTC. However, given the consistency of the 50-year cut-off across Middle Eastern and European cohorts, it may suggest a broader revision of the current AJCC threshold is warranted.

It is important to acknowledge some limitations of this study. The retrospective design from a single institute could introduce inherent biases, and the findings may not be applicable to other populations or ethnicities. Additionally, vast majority of the cohort is papillary thyroid carcinoma, with limited number of Follicular thyroid carcinoma, which prevented us from comparing the age cut-off for these subtypes of DTC individually. Additionally, while stage III and IV cases were relatively small in number, our statistical analyses were performed on the full cohort to ensure adequate power.

In conclusion, our study suggests that using an age cut-off of 50 years in the TNM staging system improves the predictability of DSS DTC patients from Middle Eastern ethnicity. Implementing age cut-offs tailored to Middle Eastern population can enhance the accuracy of prognostic assessment and ultimately lead to better patient outcomes.

## Data Availability

The original contributions presented in the study are included in the article/supplementary material. Further inquiries can be directed to the corresponding author.
